# Reference Gene Selection for Gene Expression Analyses in Mouse Models of Acute Lung Injury

**DOI:** 10.3390/ijms22157853

**Published:** 2021-07-22

**Authors:** Athanassios Fragoulis, Kristina Biller, Stephanie Fragoulis, Dennis Lex, Stefan Uhlig, Lucy Kathleen Reiss

**Affiliations:** 1 Department of Anatomy and Cell Biology, Uniklinik RWTH Aachen University, 52074 Aachen, Germany; afragoulis@ukaachen.de; 2 Department of Pharmacology and Toxicology, Uniklinik RWTH Aachen University, 52074 Aachen, Germany; kbiller@ukaachen.de (K.B.); stephie.siegl@googlemail.com (S.F.); Dennis.Lex@bfarm.de (D.L.); suhlig@ukaachen.de (S.U.)

**Keywords:** primer validation, reference gene establishment, qPCR normalisation, ALI mouse models, VILI, IPL, acid-induced lung injury, LPS-induced lung injury

## Abstract

qRT-PCR still remains the most widely used method for quantifying gene expression levels, although newer technologies such as next generation sequencing are becoming increasingly popular. A critical, yet often underappreciated, problem when analysing qRT-PCR data is the selection of suitable reference genes. This problem is compounded in situations where up to 25% of all genes may change (e.g., due to leukocyte invasion), as is typically the case in ARDS. Here, we examined 11 widely used reference genes for their suitability in commonly used models of acute lung injury (ALI): ventilator-induced lung injury (VILI), in vivo and ex vivo, lipopolysaccharide plus mechanical ventilation (MV), and hydrochloric acid plus MV. The stability of reference gene expression was determined using the NormFinder, BestKeeper, and geNorm algorithms. We then proceeded with the geNorm results because this is the only algorithm that provides the number of reference genes required to achieve normalisation. We chose interleukin-6 (*Il-6*) and C-X-C motif ligand 1 (*Cxcl-1*) as the genes of interest to analyse and demonstrate the impact of inappropriate normalisation. Reference gene stability differed between the ALI models and even within the subgroup of VILI models, no common reference gene index (RGI) could be determined. NormFinder, BestKeeper, and geNorm produced slightly different, but comparable results. Inappropriate normalisation of *Il-6* and *Cxcl1* gene expression resulted in significant misinterpretation in all four ALI settings. In conclusion, choosing an inappropriate normalisation strategy can introduce different kinds of bias such as gain or loss as well as under- or overestimation of effects, affecting the interpretation of gene expression data.

## 1. Introduction

Acute respiratory distress syndrome (ARDS) is a devastating inflammatory lung disease, affecting approximately 10% of all intensive care unit patients, whereof around 40% perish in hospital [1]. Due to SARS-CoV-2, the incidence for acute respiratory failure has increased enormously in the past year, given that classical and coronavirus disease-2019 (COVID-19)-related ARDS are closely related [2,3]. Clearly, altered gene expression plays a critical role in ARDS, and it has been estimated that 10% to 25% of all genes may change their expression in this condition [4]. This impressive number illustrates the challenge of identifying suitable reference genes, which are indispensable as a yardstick to quantify changes in gene expression.

In order to study ARDS, many different mouse models have been developed [5]. To establish a procedure for normalisation based on reference genes, here, we examined the three most commonly used acute lung injury (ALI) models—each of them addressing different aetiologies of pulmonary inflammation—in a mouse intensive care unit (MICU) that provides sedation, mechanical ventilation (MV), oxygen support, and pulmonary and cardiovascular monitoring [6].

The most frequently used one-hit model appears to be ventilator-induced lung injury (VILI) [5,7]. It causes biotrauma and, if ventilation pressure is high enough, also mechanical disruption of the alveolar–capillary barrier in healthy lungs [8]. This model helps to understand the molecular processes in ventilated ARDS patients, who can develop ventilator-associated lung injury [9]. In order to study VILI and eliminate the effects of immune cell sequestration, we used isolated perfused lungs in a well-established setup [10,11].

Another popular two-hit model is the instillation of lipopolysaccharide (LPS), a glycolipid derived from the outer membrane of Gram-negative bacteria that binds to the Toll-like receptor 4 and activates the innate immune response [12]. Although the sterile LPS-driven inflammation cannot be equated with bacterial infection, LPS is used with the attempt to model the potentially devastating immune responses of ARDS patients (cytokine storm) and to induce pulmonary inflammation in a dose-dependent matter [7].

A further important two-hit model (i.e., i.t. instillation of low pH (<2.0) hydrochloric acid (HCl)) aims to mimic gastric aspiration. Although the pulmonary consequences of aspiration are not solely related to the acidic pH, this model is highly relevant because gastric aspiration causes more than 10% of all ARDS cases [13]. Acid produces an initial chemical injury within 1 h, followed by an inflammatory response with features of human ARDS after about 3 h [14].

A general weakness of animal studies is that they often translate poorly into the clinical situation, even when performed under the highest standards. In addition, reproducibility is often hampered by variability between different labs even when similar models are used [15], possibly explained by different experimental setups, genetic animal backgrounds, assay conditions, and other factors. It is therefore of great importance to not introduce further ambiguities into the analysis.

At present, real-time quantitative reverse-transcriptase PCR is the standard tool for detection and quantification of RNA expression and is widely used to analyse inflammatory gene expression. However, despites its wide-spread use, the accuracy and reliability of qPCR depends on good and standardised laboratory practice, as outlined by Bustin and colleagues in 2009 in the MIQE guidelines [16], as much as it does on proper data analyses. Because qPCR gene expression is usually expressed in relative terms, proper normalisation of the data is of utmost importance. While it is known that the validity of these analyses depends on the stability of the reference gene(s), their selection is often not well-founded. As recently shown for a preterm lamb model, commonly used reference genes such as *18S rRNA* or *Rps29* are not necessarily the best choice for this model, suggesting that reference genes need to be determined for each model [17]. Furthermore, it seems questionable whether a single control reference is sufficient for proper normalisation, as this may lead to erroneous normalisation [18,19]. A recent reference gene validation study in a model of Duchenne muscular dystrophy has shown that gene stability changes with experimental conditions and no single gene is stable under all conditions [20]. As a solution, the use of a reference gene index, containing multiple reference genes, has already been recommended in the early 2000s [19].

This led us to the present study in which we analysed the stability of 11 reference genes (*Actb*, *B2m*, *Eef2*, *Gapdh*, *Hprt*, *Rpl13a*, *Rps29*, *Sdha*, *Tbp*, *Tubb4b*, *Ywhaz*) and their effect on data normalisation in different murine lung injury models. Although animal models and ARDS patients share many common features of inflammation, different stimuli and models also show critical differences with respect to gene expression [4,5]. Therefore, a separate reference gene stability analysis is required for each model and was performed here using three different commonly used algorithms: Bestkeeper [21], Normfinder [22], and the pairwise approach of geNorm [19]. We then chose Interleukin 6 (*Il-6*) and C-X-C motif ligand 1 (*Cxcl-1*) (as murine Interleukin 8 (*Il-8*) homologue) mediators, an cytokine well-known to play an important role in ARDS [23,24] as the exemplary target gene to examine and document the relevance of reference gene stability on gene normalisation.

Our study highlights the importance of reference gene stability analysis and shows how improper reference genes can lead to misinterpretations. Furthermore, we provide validated reference gene candidates for three popular lung injury models in mice.

## 2. Results

### 2.1. Selection of Candidate Reference Genes

To evaluate a valid and robust normalisation strategy for each experimental approach, we selected a set of 11 candidate reference genes (Table 1, reference genes/REF). To avoid any selection bias, we chose genes that (I) have already been described as potential reference genes in experimental studies and (II) that belong to different biological processes, so that model-related effects on one biological process do not impact multiple candidate genes. For that reason, we included *Actb* (beta-actin; cytoskeletal structural protein), *B2m* (beta-2 microglobulin; subunit of MHC-I complexes), *Eef2* (eukaryotic translation elongation factor 2; protein translation), *Gapdh* (glyceraldehyde-3-phosphate dehydrogenase; carbohydrate metabolism), *Hprt* (hypoxanthine guanine phosphoribosyl transferase; purine metabolism), *Rpl13a* (ribosomal protein L13A; ribosomal protein), *Rps29* (ribosomal protein S29; ribosomal protein), *Sdha* (succinate dehydrogenase complex, subunit A flavoprotein; subunit of mitochondrial complex II), *Tbp* (TATA box binding protein; basic transcription factor), *Tubb4b* (tubulin, beta 4B class IVB; constituent of microtubules), and *Ywhaz* (tyrosine 3-monooxygenase/tryptophan 5 monooxygenase activation protein, zeta polypeptide; member of 14-3-3 protein family, major regulator of apoptotic pathways critical to cell survival). Table 1 summarises the qPCR-relevant information of the related oligonucleotides including specificity control parameters such as amplicon length (via agarose gel electrophoresis) and melt temperature Tm (via melt curve analyses). Further details on primer binding sites, RNA, and PCR quality control experiments are provided in Supplementary Materials S1–S3.

### 2.2. Amplification Efficiency and General qPCR Parameters

The results of the reference gene establishment approach are summarised in Table 2. The calculated amplification factors varied between 1.85 and 2.05, and the amplification efficiency between 84.75% and 104.62%. The correlation coefficient value (R^2^) for all genes was good and varied only slightly between 0.990 and 0.999. The expression of all candidate reference genes was assayed for each experimental model.

**Figure 1:**
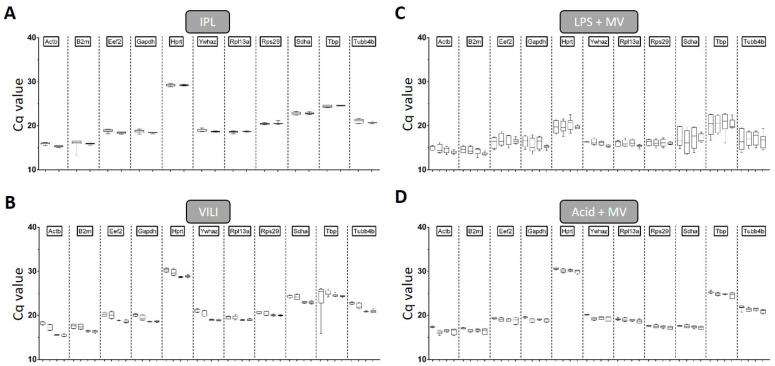
Cq distribution of candidate reference genes. Cq values for each reference gene and each treatment regimen are depicted separately. Each single graph represents the data of one experimental approach and the ticks on the *x*-axis the experimental groups: (**A**) IPL groups: NV & OV (from left to right); (**B**) VILI groups: p10, p24, p27 & p30; (**C**) LPS + MV groups: NaCl, 0.001, 0.25 and 2.5 mg LPS; (**D**) Acid + MV groups: NaCl, pH 2.0, pH 1.8 and pH 1.5; data: boxes encompass the 25th to 75th percentiles (line = median). Whisker caps denote the minimum and maximum values.

Table 3 depicts the descriptive statistics on qPCR parameters for these approaches, which revealed that the highest overall expression was found for *Actb* (Cq: 14.53 ± 0.74–16.94 ± 0.75) and *B2m* (Cq: 14.19 ± 0.72–17.05 ± 0.65), while *Hprt* was the least expressed gene across all models (Cq: 19.92 ± 1.07–29.33 ± 0.73). The Cq distribution of each gene and model is further visualised in Figure 1 (A: IPL; B: VILI; C: LPS + MV; D: Acid + MV). The coefficients of variance (%CV) of the genes investigated were comparable within the same experimental conditions, but differed between the models (Table 2) with the exception of *Actb* and *Sdha* where the CVs deviated in the in vivo flexiVent ventilation (VILI) and the in vivo flexiVent LPS instillation model (LPS + MV), respectively. Taking together the CVs of each gene for all models, the analyses showed that variability was the smallest in the ex vivo IPL ventilation model (IPL, mean: 1.41%, range: 0.62–2.82%), followed by the in vivo flexiVent acid instillation model (Acid + MV, mean: 2.83%, range: 1.83–4.43%), in vivo flexiVent ventilation model (VILI, mean: 3.61%, range: 1.82–7.35%), and the in vivo flexiVent LPS instillation model (LPS + MV, mean: 6.67%, range: 3.90–12.01%).

### 2.3. Normalisation Strategies

**Figure 2:**
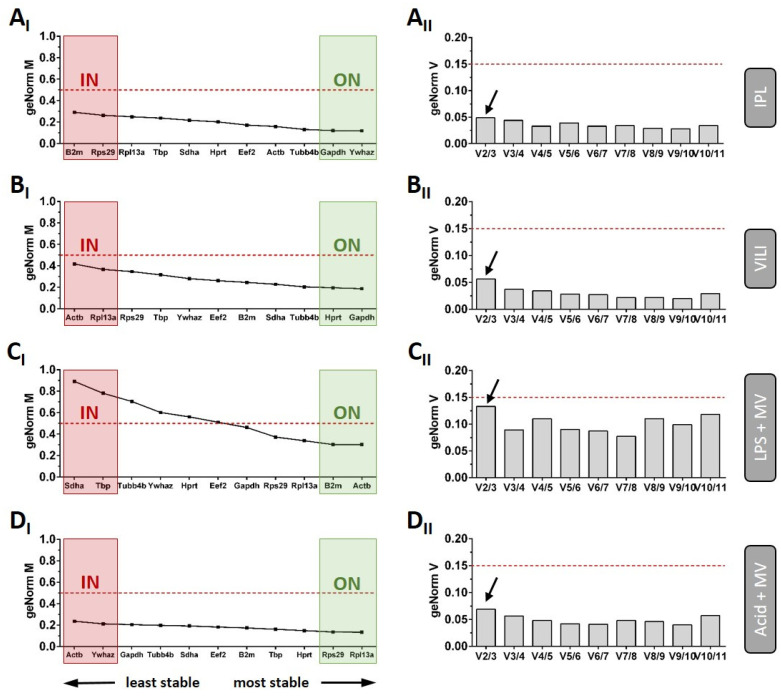
Results from the geNorm analyses. geNorm M-values (subscript I) and V-values (subscript II) were calculated and are depicted for each experimental approach separately. geNorm M results are shown as ranking, indicating the stability from left to right as least to most stable genes. Cut-off values for both analyses are indicated by red dotted lines. The optimised (ON) as well as the most inappropriate normalisation (IN) strategy are highlighted in the graphs with green and red boxes, respectively. (**A_I_**) IPL geNorm results revealed *Ywhaz* and *Gapdh* as most as well as *B2m* and *Rps29* as the least stable reference genes, (**B_I_**) VILI geNorm results revealed *Gapdh* and *Hprt* as most as well as *Actb* and Rpl1*3a* as the least stable reference genes, (**C_I_**) LPS + MV geNorm results revealed *Actb* and *B2m* as most as well as *Sdha* and T*bp* as the least stable reference genes. (**D_I_**) Acid + MV geNorm results revealed *Rpl13a* and *Rps29* as most as well as *Actb* and *Ywhaz* as the least stable reference genes. (**A_II_**–**D_II_**) Based on the geNorm V analysis, two reference gene are sufficient for a reliable normalisation in all investigated models (arrows: V-value of the V2/3 comparison all < 0.15).

The three most popular algorithms (BestKeeper, NormFinder, and geNorm) were initially applied to determine reference gene stabilities and rankings for each model separately. The combined ranking represents the geometric mean of the single rankings of these algorithms (Tables S3–S6). The analyses showed that, apart from the LPS model, the reference gene stabilities were generally high, with rather small differences between the individual genes. On the other hand, the calculated rankings of BestKeeper, NormFinder, and geNorm differed noticeably, which might be explained by the different algorithms. Because of that, we decided not to use the combined ranking to specify the optimal normalisation strategies, but prioritised the most reliable algorithm. Since geNorm is the only algorithm that not only provides stability rankings (M-value), but also determines the minimal number of reference genes (V-value) to use in the reference gene index (Figure 2 A: IPL, B: VILI, C: LPS + MV & D: Acid + MV), we decided to use this program for further analysis of our data. The red dotted lines in Figure 2 represent the pre-defined standard cut-off values for these analyses (M-value: 0.5; V value: 0.15). It can be seen that none of the genes exceeded the M-value cut-off in A, B, and D, which demonstrates the high overall stability in these models (mean + SD M-values: IPL = 0.197 + 0.060, VILI = 0.278 + 0.076, Acid + MV = 0.180 + 0.033). As depicted in Figure 2C_I_, LPS instillation combined with mechanical ventilation, however, was characterised by decreased gene stabilities, with six genes even exceeding the critical cut-off M-value of 0.5 (mean + SD M-value: IPL = 0.530 + 0.200). Nevertheless, V-value calculations revealed that a reference gene index including the two most stable reference genes was sufficient for optimal normalisation in all models (Figure 2, all geNorm V-values < 0.15; arrows), even in the LPS model. In addition, the most inappropriate normalisation strategy was defined to include the two least stable genes for each approach. The corresponding gene pairs are color-coded in the M-value graphs of Figure 2 and in Tables S1–S4 (green: optimized normalisation ON, red: inappropriate normalisation IN).

### 2.4. Comparison of IL-6 and Cxcl1 Gene Expression Using an Optimised vs. Inappropriate Normalisation Strategy

**Figure 3:**
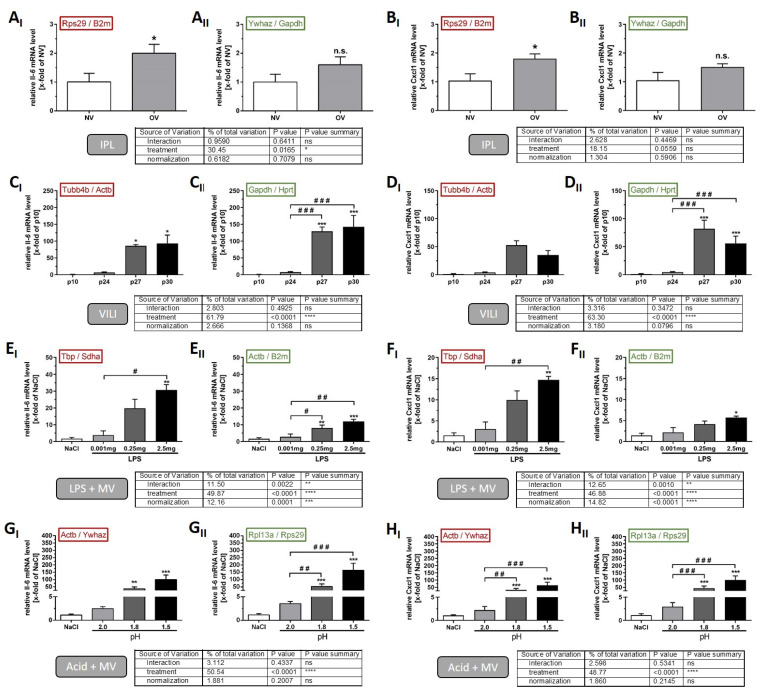
Comparative gene expression analyses of *Il-6* and *Cxcl1* applying an inappropriate or optimised normalisation strategy according to the geNorm results. The experimental models were conducted as described in the M&M section. The *Il-6* and *Cxcl1* gene expression levels in the (**A**,**B**) IPL, (**C**,**D**) the VILI, (**E**,**F**) the LPS + MV, and (**G**,**H**) the Acid + MV approach were determined by normalisation using either the least appropriate (figure caption with subscripted **I**) or the optimised (figure caption with subscripted **II**) reference gene index. Statistical significances are indicated as * *p* < 0.05, ** *p* < 0.01, *** *p* < 0.001 vs. control groups (IPL: NV, VILI: p10, LPS & Acid + MV: NaCl); # *p* < 0.05, ## *p* < 0.01, ### *p* < 0.001 as indicated by the brackets. The tables below the graphs show the individual metrics for comparison of the optimised versus the least appropriate normalisation strategy for each model, with the following significances ** *p* < 0.01, *** *p* < 0.001, **** *p* < 0.0001 or n.s. (not significant).

To demonstrate the relevance of the described way to establish reference genes, we conducted representative analyses with all models comparing the optimised normalisation with the least appropriate normalisation strategy. For this purpose, we measured the gene expression of *Il-6* and *Cxcl1* (Table 1, genes of interest GOI), since they both represent relevant mediators in all conducted ALI models. As the geNorm analyses suggested, a normalisation by a RGI based on the geometric mean of two genes, we applied that for both conditions IN (Figure 3, I-graphs) as well as ON (Figure 3, II-graphs). Data on amplification efficiency calculation for each approach and gene are depicted in Table 4.

The ex vivo IPL setup contained ventilation procedures with low (NV = 8 cmH_2_O) and high pressure (OV = 25 cmH_2_O). Both *Il-6* and *Cxcl1* gene expression was only slightly induced by OV and the mean values did not differ statistically between the normalisation strategies (*Il-6*: Figure 3A, ON: 1.6-fold and IN: 2-fold induction compared to NV, normalisation factor *p* = 0.7079 and *Cxcl1*: Figure 3B, ON: 1.5-fold and IN: 1.8-fold induction compared to NV, normalisation factor *p* = 0.5906). However, for both genes, this induction of gene expression compared to control conditions was only statistically significant when the data were normalised to the inappropriate reference gene index (Figure 3(A_I_,B_I_), red: *Rps29/B2m*), while that was not the case when normalised to the optimised RGI (Figure 3(A_II_,B_II_), green: *Ywhaz/Gapdh*).

In our second experimental approach, the single-hit in vivo ventilation model (VILI), we applied four different ventilation pressures (p10, p24, p27, and p30), of which the lowest was comparable to the NV strategy in the IPL setting, whereas the higher pressures were variants of overventilation, which are at the threshold between almost no injury and lethal lung failure in vivo [8]. Depending on the normalisation strategy, the gene expression levels of both *Il-6* and *Cxcl1* differed in terms of loss of statistical significances/effects as depicted in Figure 3C,D (loss of differences between p24 and p27/p30), thereby leading to a significant misinterpretation of inter-group effects, if not normalised appropriately. Moreover, the induction of gene expressions for both genes were generally lower if normalised to the inappropriate (Figure 3(C_I_,D_I_), red: *Tubb4b/Actb*) instead of the optimised RGI (Figure 3(C_II_,D_II_), green: *Gapdh/Hprt*). The relative expression of *Il-6* decreased from 8- to 6-fold of p10 after p24 ventilation, from 130- to 85-fold of p10 after p27, and from 143- to 93-fold of p10 after p30 ventilation. Similar to this, the relative gene expression of *Cxcl1* decreased from 5-to 4-fold of p10 after p24 ventilation, from 82- to 56-fold of p10 after p27, and from 143-to 35-fold of p10 after p30 ventilation in this context. However, statistical significances of inter-group comparisons were not affected by the different RGI.

We also included two different 2-hit in vivo models in our study: LPS-instillation and acid-instillation with subsequent ventilation-induced lung injury. LPS instillation resulted in an increase of both *Il-6* and *Cxcl1* gene expression compared to the NaCl control treatment, regardless of which RGI was used for data analysis. However, the results were clearly affected by the use of the inappropriate RGI (Figure 3(E_I_,F_I_), red: *Tbp/Sdha*) compared to the optimised RGI (Figure 3(C_II_,D_II_), green: *Actb/B2m*), as indicated by a significant normalisation effect (Figure 3E, table: normalisation factor Il-6 *p* = 0.0001; Figure 3F, table: normalisation factor *Cxcl1 p* < 0.0001) in the analysis. Inappropriate normalisation by *Tbp/Sdha* increased the relative gene expression of *Il-6* from 2.8- to 3.9-fold after 0.001 mg LPS, from 8.1-to 19.8-fold after 0.25 mg LPS and from 12.1- to 30.7-fold after 2.5 mg LPS instillation compared to the NaCl control. The relative expression levels of *Cxcl1* were affected similarly by inappropriate normalisation as they increased from 2.2-to 3.1-fold after 0.001 mg LPS, from 4.2- to 10.0-fold after 0.25 mg LPS, and from 5.7- to 14.7-fold after 2.5 mg LPS instillation compared to the NaCl control. The three main issues associated with IN in this model were the overestimation of *Il-6* and *Cxcl1* induction due to increased effect expression, loss of statistical significance due to increased biological variability, and, associated with this, a loss of concentration dependence of the LPS effect in the case of *Il-6* gene expression.

The analyses of the *Il-6* expression data from the in vivo acid instillation model revealed significant differences between the applied normalisation strategies. The instillation of acid with either pH 1.8 or pH 1.5 resulted in a significant induction of *Il-6* gene expression, but without noticeable differences in mean values between normalisation strategies (Figure 3D, table: normalisation factor *p* = 0.2007). However, while the inter-group comparison between pH 2.0 and the two other treatment groups was significantly different if data were normalised to the optimised RGI (Figure 3D_II_, green: *Rpl13a/Rps29*), these comparisons did not reach statistical significance in the case of unsuitable normalisation (Figure 3D_I_, red: *Actb/Ywhaz*). In contrast, for *Cxcl1* gene expression analysis, we detected no differences between the two normalisation strategies, neither for mean values nor for statistical effects (Figure 3G,H).

## 3. Discussion

RT-qPCR is widely used for gene expression studies due to its high sensitivity and specificity at relatively low cost. Therefore, even with next generation sequencing on the rise, it remains the most widely used method for gene expression analysis of specific biomarkers in molecular biology research. To circumvent possible variations in RNA extraction/yield and reaction efficiency and to compare different experimental conditions, as exemplified in our study, accurate normalisation by appropriate reference genes is of crucial importance. While expression levels of commonly used reference genes are known to vary between different cells, tissues, and conditions, a lack of evidence for usefulness, normalisation by *Actb* and *Gapdh* is frequently relied upon in RT-qPCR studies without further validation, not only in lung research, but generally in vertebrates [25]. This might have historical reasons or could be due to the fact that these genes have been shown to be stably expressed in specific settings, even though several studies on reference gene stability in the lung show that the suitability of reference genes in lung models cannot be generalised. The stability of *Actb*, *Gapdh* and other frequently used reference genes (e.g., *Rps29*, *18s RNA*, *Ywhaz*, *Sdha*, and *Hprt*) varies strongly in the lung and these candidates can turn out as the best choice, but might just as well be not suitable at all [17,26,27,28,29]. For that reason, we analysed a set of eleven candidate reference genes for their suitability to normalise gene expression data obtained from four commonly used experimental approaches in mice in the field of ARDS research [5].

In line with the studies above-mentioned, our comparison confirmed that the suitability of a commonly used ‘housekeeper’ cannot be assumed per se. More precisely, in our acid model, *Gapdh* and *Actb* were amongst the three least stable genes. In the LPS model, *Gapdh* also proved not to be stable, whereas *Actb* turned out to be the most stable gene. Vice versa, in the in vivo ventilation model, in which inflammation is less profound than with acid or LPS, *Gapdh* was the most stable and *Actb* the least stable gene. Our findings correlate with biological functions of these genes. Since *Actb* is one of the major components of the cytoskeleton with a role in mechanotransduction [30], it is reasonable that it is particularly regulated in models with pronounced mechanical stress. *Gapdh*, on the other hand, has frequently been shown to be strongly regulated in the course of metabolic reprogramming that occurs under inflammatory conditions [31], as present in our Acid + MV and LPS + MV models. Regarding the majority of tested genes, the picture was completely different again in the IPL ventilation setting and our data illustrated a similar variability between the four settings for the other tested genes. Our findings clearly demonstrate that an inappropriate normalisation has significant effects on the results and the interpretation of the data, even under conditions where reference gene expression is stable, as indicated by the low M-value in three of the four experimental approaches. Thus, our study demonstrates the significant dimension of bias that can be introduced at this point.

In order to compare normalisation with the least (IN) versus the most suitable (ON) RGI, we chose two exemplary pro-inflammatory mediators: *Il-6* and *Cxcl1*, which is a murine *Il-8* homologue. Il-6 and Il-8 levels correlate with tidal volume and mortality in human ARDS, as already shown over 20 years ago [32,33,34]. They are equally important in experimental ALI, and belong to the standard readout parameters in most ALI studies [6,8,35,36,37]. While Il-6 is a cytokine that elicits inflammation and, moreover, cytokine storm [24], the chemokine Il-8 is co-responsible for neutrophil recruitment, a hallmark of ARDS/ALI pathogenesis [38]. In two recent phenotyping approaches of human ARDS, Il-6 and Il-8 have been found to be predictive for the hyperinflammation and the reactive phenotype, both correlating with higher mortality than the respective phenotype with lower Il-6 and Il-8 [39,40]. Furthermore, these two mediators are likewise relevant in COVID-19 induced ARDS [41].

We were able to show that this normalisation-mediated bias would have led to crucial misinterpretation of the *Il-6* and *Cxcl1* data in our models. We observed four distinct issues of inappropriate normalisation in our experiments—loss or gain of statistically significant effects as well as under- or overestimation of effect magnitudes. This is not surprising, considering that reference gene stability can even vary in the same model, when performed under different conditions [25]. Interestingly, the extent of statistical bias was associated with the overall gene stability in the models. While the more stable models were less susceptible, analysis of the data from the LPS model, where there was a marked difference in stability between IN and ON (mean M-value = 0.530), actually showed a combination of both spurious effects (loss of effects plus underestimation of effect magnitude). Of note, only in one out of eight analyses (Figure 3H, *Cxcl1* Acid + MV), inappropriate normalization did not influence data interpretation. Therefore, our study nicely demonstrates how normalisation against inappropriate reference genes may result in small but potentially biologically relevant effects being undetected or misinterpreted. In practice, inadequate normalisation can lead to a wrong interpretation of an ALI model and even to false conclusions regarding the tested interventions. This is particularly relevant when gene expression analysis is not complemented by protein quantification data. On the other hand, many studies show that gene and protein expression do not always accord well [42]. Besides the regulatory mechanisms and the time point of sample collection, this might also be explained by the common utilisation of inappropriate reference genes. Obviously, these findings should also be relevant for gene expression studies in humans with COVID-19.

## 4. Materials and Methods

### 4.1. Animals and Sample Collection

Female C57BL/6 mice (8–12 weeks, weighing 20–25 g) were kept under standard conditions. All experiments were in accordance with the German animal protection law and European Directive 2010/63/EU and approved by regional governmental authorities (LANUV NRW, permission numbers: AZ84-02.04.2013.A078, AZ84-02.04.2015.A385, AZ84-02.04.2013.A131, and 10509A6).

Three ALI models were carried out: the one-hit VILI model, in vivo and ex vivo in isolated perfused lungs, the two-hit model of acid-induced lung injury and MV (Acid + MV), and the two-hit model of lipopolysaccharide induced lung injury and MV (LPS + MV). Surgical procedures were performed as described [6]. In brief, mice were initially anaesthetised with pentobarbital sodium (75 mg/kg) and fentanyl (40 µg/kg), followed by tracheotomy with a 20-gauge cannula and connect to the respective ventilator. A catheter for blood pressure monitoring and permanent infusion of 0.9% NaCl (200 µL/h) was inserted into the carotid artery. Blood pressure and ECG were recorded permanently (PowerLab, ADInstruments, Spenbach, Germany). In addition, pulsoxymetry was performed with a tail clip (MouseOx, STARR Life-Science, Oakmont, PA, USA). Body temperature was maintained between 36.5 °C and 37.5 °C by a homeothermic blanket (Harvard Apparatus Holliston, MA, USA).

Table S1 gives an overview of the model details of the in vivo models. The samples from the in vivo and ex vivo models, used in this study, were generated in previous projects, as cited below. In addition, successful induction of ALI is shown here by the Horovitz index (Table S2).

In the VILI model, plateau pressures of 10 cmH_2_O (p10), 24 cmH_2_O (p24), 27 cmH_2_O (p27), and 30 cmH_2_O (p30) were used as described previously. Ex vivo ventilation experiments (IPL) were also performed as described before [43]. After baseline recording with *p* = 8 cmH_2_O, f = 90 min^−1^, and PEEP = 3 cmH_2_O, ventilation was continued for 210 min, with high (*p* = 25 cmH_2_O; OV = over ventilation) versus low pressure (*p* = 8 cmH_2_O, NV = normal ventilation).

In the model Acid + MV, mice received 50 µL hydrochloric (HCl) with pH = 2.0, pH = 1.8, or pH = 1.5. MV with the flexiVent ventilator (SCIREQ, Montreal, Canada) was performed with VT = 16 mL/kg, f = 80 min^−1^, PEEP = 2 cmH_2_O and FiO_2_ = 0.3 and was terminated after 330 min [6,44]. In the model LPS + MV, mice were instilled with 0.001 mg/kg, 0.25 mg/kg, or 2.5 mg/kg LPS (LPS *Escherichia coli* O111-5MG, Lot #127M4016V, Sigma, St. Louis, MO, USA) and were ventilated as in the acid model, but for 7 h [45]. In both models, controls were instilled with 0.9% NaCl.

### 4.2. RNA Isolation, cDNA Synthesis, and qPCR

For gene expression analysis, RNA was isolated fully automated with the QIAcube (QIAGEN GmbH; Hilden, Germany) using the RNeasy^®^ Mini Kit (QIAGEN GmbH; Hilden, Germany). Therefore, snap-frozen lung tissue (15 mg) was ground in liquid nitrogen followed by homogenisation using the Precellys^®^ Tissue Homogeniser. RNA concentration and purity was determined spectrophotometrically with the NanoDrop (Peqlab; Erlangen, Germany). The integrity of RNA was assessed either by agarose gel electrophoresis or by the Agilent 2100 Bioanalyzer (Agilent Technologies, Santa Clara, CA, USA) displaying RNA integrity as the RNA integrity number (RIN) (Figure S1). One µg of total RNA were transcribed into cDNA using the Maxima Reverse Transcriptase (RT) (Thermo Fisher Scientific; Waltham, MA, USA) and mixed priming oligo-(dT)18:random hexamer ratio of 3:1, *v*/*v*), according to the manufacturer’s instructions with 15 min RT incubation.

PCR reactions took place in a total volume of 10 µL containing 1 µL cDNA (equates to 25 ng cDNA), 5 µL SYBR-Green Mastermix I (Roche-Diagnostics GmbH, Mannheim, Germany), 3 µL Nuclease-free water, 0.5 µL each of a forward primer (10 µM), and the corresponding reverse primer (10 µM). After heat activation of the polymerase at 95 °C for 5 min, every cycle consisted of denaturation of the double-stranded DNA template at 95 °C for 10 s, annealing of the primers to the single-stranded DNA templates at specific temperature for 10 s, and elongation at 72 °C for 15 s. The target genes *Il-6* and *Cxcl1* were subjected to a different cycle protocol with the following conditions: single heat activation at 95 °C for 10 min and 40 times 95 °C for 15 s; specific annealing temperature for 30 s and 72 °C for 1 min.

Quantitative PCR was performed in technical triplicates. Reactions were run on a LightCycler^®^ 480 System (Roche, Basel, Switzerland) for 40 cycles using SYBR Green I technology. Amplification efficiency was calculated by slope analyses of standard curves prepared from a defined cDNA-dilution series within each dataset or with LinReg 2016.0 software (Heart Failure Research Centre, Amsterdam, The Netherlands) as described before [46]. More details are provided in the Supplementary Materials.

### 4.3. Primer Design

Primers were created with the online primer design tool Primer-BLAST from NCBI (see Figure 2 for primer binding sites), and the physiochemical properties of the oligonucleotides (amplification length and T_m_, secondary structures of amplicon, primer-dimer formation) were calculated using OligoAnalyser 3.1 (Integrated DNA Technologies; Leuven, Belgium) and OligoCalc (http://biotools.nubic.northwestern.edu/OligoCalc.html, (accessed on 21 July 2021)). The online tool uMelt (https://www.dna-utah.org/umelt/quartz/um.php, (accessed on 21 July 2021)) was used for in silico melt curve analysis. Appropriate primer annealing temperature was established by gradient qPCR. Primer specificity was validated by melt curve analysis and DNA agarose gel electrophoresis.

### 4.4. Data Analysis

The evaluation of reference gene stabilities was achieved using three validated algorithms: BestKeeper [21], NormFinder [22], and geNorm [19], each with unique advantages (details in Supplementary Materials). BestKeeper calculates the standard deviation (SD) of each gene and the geometric mean of candidate Cq values (= BestKeeper index) and, in contrast to NormFinder and geNorm, uses raw data (Cq values) instead of relative quantities [21]. Genes with the lowest SD and highest coefficient of correlation with the BestKeeper index represent the most stable reference genes.

NormFinder is a model-based approach in which both, intra- as well as intergroup variations, are considered to avoid systematic error [22].

The advantage of geNorm over the two other algorithms is the additional calculation of the number of genes required for reliable normalisation by using pairwise variation of calculated SD of the expression ratio of two reference genes for the evaluation of stabilities [19]. Since this is in line with the currently most accepted normalisation procedures using a reference gene index (RGI) containing multiple reference genes instead of single gene normalisation, we used the geNorm results for our study. The relative changes in *Il-6* and *Cxcl1* gene expression normalised to either the most stable or least stable RGI (based on geNorm results) was calculated for each mouse model. In geNorm, the gene-stability measure (called M value) is calculated for each measured gene and represents the average pairwise variation of the specific gene compared to all the other reference genes. Higher M values correlate with low and smaller M values with high expression stability.

Each analysis was conducted with 12 samples for ex vivo IPL, 15 samples for the in vivo models, and 11 reference gene candidates. For the target genes, we used *n* = 6 in the Acid + MV and in the LPS + MV model and *n* = 5 in the IPL setting. In the VILI study, the following sample numbers were available: *n* = 3 in the group p10, *n* = 5 in the groups p24 and p27, and *n* = 6 in the group p30.

### 4.5. Statistics

Variance homogeneity was checked with the Bartlett test. The Shapiro–Wilk test was used to test for normal distribution. BoxCox-Y transformation was conducted to achieve homoscedasticity if necessary. One-way ANOVA followed by the Tukey post-hoc test was performed to analyse the parametric data. Non-parametric data were analysed using the Kruskal–Wallis test followed by Dunn’s post-hoc testing. Data are shown as mean + SEM. Statistical analyses were performed with GraphPad Prism 9 (GraphPad, La Jolla, CA, USA) and JMP 10 (Böblingen, Germany).

## 5. Conclusions

In line with the current recommendations on qPCR, our study underlines the necessity to test the stability of reference genes in every specific setting. Nevertheless, this might not be feasible in every laboratory. For this case, our study suggests suitable normalisation strategies for the three most commonly used ALI models in mice and provides the necessary validated primer sequences.

## Figures and Tables

**Table 1:** Data on applied primer sets used to measure the reference genes (REF) and the gene of interest (GOI).

Target Type	Gene	Accesion Number	Primer Sequence 5′′→3′′	Annealing Temp [°C]	Amplicon Length [bp]	Tm [°C]
REF	*Actb*	NM_007393.5	F: CAC TGT CGA GTC GCG TCC	60	89	88.20
			R: TCA TCC ATG GCG AAC TGG TG			
	*B2m*	NM_009735.3	F: TTC TGG TGC TTG TCT CAC TGA	61	104	83.27
			R: CAG TAT GTT CGG CTT CCC ATT C			
	*Eef2*	NM_007907.2	F: TCA CAA TCA AAT CCA CCG CC	60	122	83.87
			R: ATG GCC TGG AGA GTC GAT GA			
	*Gapdh*	NM_008084.3	F: CAT GGC CTT CCG TGT TCC TA	60	74	85.63
			R: ACT TGG CAG GTT TCT CCA GG			
	*Hprt*	NM_013556.2	F: TCA GTC AAC GGG GGA CAT AAA	61	142	79.10
			R: GGG GCT GTA CTG CTT AAC CAG			
	*Rpl13a*	NM_009438.5	F: GCGGATGAATACCAACCCCT	61	179	90.19
			R: CCACCATCCGCTTTTTCTTGT			
	*Rps29*	NM_009093.2	F: CCTTTCTCCTCGTTGGGCG	61	105	87.52
			R: GAGCAGACGCGGCAAGAG			
	*Sdha*	NM_023281.1	F: GGAACACTCCAAAAACAGACCT	60	106	80.41
			R: CCACCACTGGGTATTGAGTAGAA			
	*Tbp*	NM_013684.3	F: ATCTACCGTGAATCTTGGCTGT	61	183	82.21
			R: GATTGTTCTTCACTCTTGGCTC			
	*Tubb4b*	NM_146116.2	F: TCTTCTACAGCTGTTCCGCAG	61	143	89.73
			R: GTGGTAAGTGCCAGTGGGAT			
	*Ywhaz*	NM_011740.3	F: GAAAAGTTCTTGATCCCCAATGC	62	134	82.18
			R: TGTGACTGGTCCACAATTCCTT			
GOI	*Cxcl1*	NM_008176.3	F: CAAACCGAAGTCATAGCCAC	60	106	83.10
			R: TGGGGACACCTTTTAGCATC			
	*IL-6*	NM_031168.1	F: TGCAAGAGACTTCCATCCAGTTGCC	59	147	84.92
			R: AAGCCTCCGACTTGTGAAGTGGT			

**Table 2:** Results of the PCR amplification efficiency calculations of the validation study.

Gene	Amplification Factor	Efficiency [%]	R^2^	Slope	Y-Intercept
*Actb*	2.010	101.01	0.994	−3.298	21.475
*B2m*	2.027	102.69	0.999	−3.259	22.111
*Eef2*	2.021	102.08	0.998	−3.273	24.188
*Gapdh*	2.012	101.22	0.999	−3.293	24.408
*Hprt*	1.850	85.03	0.990	−3.742	36.555
*Rpl13a*	1.847	84.75	0.998	−3.751	25.222
*Rps29*	2.036	103.58	0.996	−3.239	23.730
*Sdha*	1.958	95.83	0.992	−3.426	28.847
*Tbp*	2.046	104.62	0.993	−3.216	30.321
*Tubb4b*	1.923	92.28	0.995	−3.522	27.734
*Ywhaz*	2.006	100.58	0.996	−3.308	24.772

**Table 3:** PCR parameters of the reference genes derived from their qPCR (each model separately).

	IPL			VILI			LPS + MV			Acid + MV		
Gene	Mean Cq	SD	CV [%]	Mean Cq	SD	CV [%]	Mean Cq	SD	CV [%]	Mean Cq	SD	CV [%]
*Actb*	15.61	0.35	2.24%	16.46	1.21	7.35%	14.53	0.74	5.09%	16.94	0.75	4.43%
*B2m*	15.97	0.45	2.82%	16.89	0.64	3.79%	14.19	0.72	5.07%	17.05	0.65	3.81%
*Eef2*	18.60	0.32	1.72%	19.37	0.84	4.34%	16.61	1.03	6.20%	19.52	0.66	3.38%
*Gapdh*	18.62	0.29	1.56%	19.11	0.68	3.56%	15.78	0.96	6.08%	19.29	0.51	2.64%
*Hprt*	29.24	0.18	0.62%	29.33	0.73	2.49%	19.92	1.07	5.37%	26.21	0.48	1.83%
*Rpl13a*	18.67	0.13	0.70%	19.20	0.35	1.82%	15.91	0.62	3.90%	19.09	0.41	2.15%
*Rps29*	20.50	0.16	0.78%	20.25	0.38	1.88%	16.10	0.63	3.91%	18.55	0.33	1.78%
*Sdha*	22.83	0.24	1.05%	23.54	0.74	3.14%	16.98	2.04	12.01%	23.29	0.61	2.62%
*Tbp*	24.50	0.19	0.78%	24.85	0.54	2.17%	20.39	1.73	8.48%	24.89	0.56	2.25%
*Tubb4b*	20.91	0.36	1.72%	21.69	0.92	4.24%	16.94	1.59	9.39%	21.44	0.65	3.03%
*Ywhaz*	18.86	0.30	1.59%	19.76	0.98	4.96%	15.80	1.24	7.85%	20.10	0.65	3.23%

**Table 4:** Results of the PCR amplification efficiency calculations of the Il-6 and Cxcl1 gene expression studies.

	IPL				VILI				LPS + MV				Acid + MV			
Gene	Efficiency	R^2^	Slope	Y-Intercept	Efficiency	R^2^	Slope	Y-Intercept	Efficiency	R^2^	Slope	Y-Intercept	Efficiency	R^2^	Slope	Y-Intercept
*Actb*					2.008	0.997	−3.304	21.531	1.974	0.999	−3.386	21.637	1.984	0.999	−3.362	22.172
*B2m*	2.027	0.999	−3.259	22.111					1.982	0.998	−3.367	21.278				
*Eef2*													2.021	0.998	−3.273	24.188
*Gapdh*	2.012	0.999	−3.293	24.408	2.039	0.996	−3.232	24.428								
*Hprt*					1.918	0.994	−3.535	35.044								
*Rpl13a*													1.847	0.998	−3.751	25.222
*Rps29*	2.036	0.996	−3.239	23.730												
*Sdha*									1.958	0.992	−3.426	28.847	1.996	0.996	−3.331	28.245
*Tbp*									2.046	0.993	−3.216	30.321				
*Tubb4b*					1.923	0.995	−3.522	27.734								
*Ywhaz*	2.006	0.996	−3.308	24.772												
*Cxcl1*	1.948	0.992	−3.452	26.908	1.943	0.996	−3.466	27.398	1.887	0.998	−3.627	25.379	1.961	0.997	−3.418	27.915
*Il-6*	1.965	0.999	−3.409	24.709	1.965	0.996	−3.410	25.633	1.922	0.996	−3.523	25.332	2.005	0.998	−3.310	28.257

Table colors: green = ON REF, red = IN REF, blue = GOI.

## Data Availability

All data are shown in the main manuscript and in the Supplementary Materials.

## Supplementary Materials

The following are available online at https://www.mdpi.com/article/10.3390/ijms22157853/s1.

## Abbreviations

Actb	beta-actin
ALI	acute lung injury
ARDS	acute respiratory distress syndrome
B2m	beta-2 microglobulin
Cq	cycle of quantification
Cxcl1	C-X-C motif ligand 1
Eef2	eukaryotic translation elongation factor 2
Gapdh	glyceraldehyde-3-phosphate dehydrogenase
HCl	hydrochloric acid
Hprt	hypoxanthine guanine phosphoribosyl transferase
Il-6	Interleukin 6
Il-8	Interleukin 8
IN	inappropriate normalisation
IPL	isolated perfused lung
NV	normal ventilation
MV	mechanical ventilation
ON	optimized ventilation
OV	overventilation
RGI	reference gene index
RIN	RNA integrity number
Rpl13a	ribosomal protein L13A
Rps29	ribosomal protein S29
Sdha	succinate dehydrogenase complex, subunit A flavoprotein
Tbp	TATA box binding protein
Tubb4b	tubulin, beta 4B class IVB
VILI	ventilator-induced lung injury
Ywhaz	tyrosine 3-monooxygenase/tryptophan 5 monooxygenase activation protein zeta
