# Supplementary material for: Reference Gene Selection for Gene Expression Analyses in Mouse Models of Acute Lung Injury
Source: Int J Mol Sci. 2021 Jul 22;22(15):7853. doi: 10.3390/ijms22157853 (PMC8346155; doi:10.3390/ijms22157853)
Supplement: Supplementary file 1 [file ijms-22-07853-s001.zip › ijms-1230843-Supplementary_IJMS_Revision-new.pdf]

Supplements

Reference gene selection for gene expression analyses in mouse models of acute lung injury

Athanassios Fragoulis, Kristina Biller, Stephanie Fragoulis, Dennis Lex, Stefan Uhlig, Lucy Kathleen Reiss

Tables

Table S1: Experimental groups

	VILI	IPL	LPS + MV	Acid + MV	LPS / Acid + MV Controls
**Instillation i.t.**	-	-	LPS (2.5, 0.25, 0.001 mg/kg)	HCl (pH 1.5, 1.8, 2)	NaCl
**P (cmH_2O)**	10 *vs* 24, 27, 30	8 *vs* 25	n.a.	n.a.	n.a.
**V_T (mL/kg)**	-	-	16	16	16
**f (min^{-1})**	90 *vs* 180	90	80	80	80
**FiO_2**	0.3	-	0.3	0.3	0.3
**EEP (cmH_2O)**	+2	-3	+2	+2	+2
**MV (h)**	7	3.5	7	5.5	7 / 5.5
**n**	3-6*	5	6	6	6

The table shows model specific experimental settings: intratracheal instillation, tidal volume (V_T), fraction of inspired oxygen (FiO_2), end-expiratory pressure (EEP), mechanical ventilation (MV). The following settings are included: ventilator-induced lung injury (VILI), isolated perfused lung (IPL), Lipopolysaccharide (LPS) + MV and Acid + MV. LPS and acid were instilled i.t. in a total volume of 50 µL. Control groups received 50 µL 0.9 % NaCl i.t.. MV was started directly after instillation. * In the original VILI study, n=6 animals were investigated (Lex and Uhlig, 2017; Anesthesiology126(5):909-922). For the present reference gene study, we used the reaming material: p10: n=3, p24: n=5, p27: n=5 and p30: n=6.

Table S2: Horovitz index (pO_2/FiO_2)

Modell	Group 1	Group 2	Group 3	Group 4
**VILI**	**P = 10 mmHg**	**P = 24 mmHg**	**P = 27 mmHg**	**P = 30 mmHg**
mmHg ± SD	529 ± 19	557± 24	166 ± 34	144 ± 11
**LPS + MV**	**NaCl**	**LPS 0.001 mg/kg**	**LPS 0.25 mg/kg**	**LPS 2.5 mg/kg**
mmHg ± SD	449 ± 76	313 ± 46	234 ± 50	191 ± 25
**Acid + MV**	**NaCl**	**HCl pH=2**	**HCl pH=1.8**	**HCl pH=1.5**
mmHg ± SD	487 ± 35	468 ± 60	296 ± 40	202 ± 50

The table shows the Horovitz index in the three analyzed *in vivo* ALI models: ventilator-induced lung injury (VILI), Lipopolysaccharide (LPS) + MV, Acid+MV. All animals were ventilated with an FiO_2=0.3. pO_2 was measured by blood gas analysis in arterial blood before termination of the respective experiment. Mean values with standard deviation (SD) are shown for each group with VILI: n=5-6, LPS + MV: n=6, Acid + MV= n=6. Interpretation of the Horovitz index: < 300mmHg mild ALI, < 200mmHg moderate ALI.

Table S3: Stability values and rankings for the IPL study

	combined ranking		BestKeeper		NormFinder		geNorm	
gene	geometric mean of ranking value	rank	stability value	rank	stability value	rank	M value	rank
Ywhaz	3.175	2	0.262	8	0.138	4	0.089	1
Gapdh	3.302	3	0.249	6	0.137	3	0.089	2
Tubb4b	5.739	7	0.302	9	0.219	7	0.131	3
Actb	7.343	10	0.315	11	0.261	9	0.160	4
Eef2	5.593	6	0.261	7	0.186	5	0.173	5
Hprt	2.621	1	0.146	3	0.112	1	0.201	6
Sdha	4.121	4	0.194	5	0.135	2	0.218	7
Tbp	5.769	8	0.154	4	0.198	6	0.238	8
Rpl13a	4.160	5	0.089	1	0.227	8	0.250	9
Rps29	5.848	9	0.132	2	0.282	10	0.264	10
B2m	10.656	11	0.311	10	0.373	11	0.292	11

Table S4: Stability values and rankings for the 1-hit VILI study

	combined ranking		BestKeeper		NormFinder		geNorm	
gene	geometric mean of ranking value	rank	stability value	rank	stability value	rank	M value	rank
Gapdh	1.710	1	0.611	5	0.096	1	0.170	1
Hprt	3.302	2	0.649	6	0.146	3	0.170	2
Sdha	3.476	3	0.652	7	0.116	2	0.222	3
B2m	4.000	4	0.541	4	0.185	4	0.233	4
Tbp	4.718	7	0.442	3	0.333	7	0.284	5
Rps29	4.579	6	0.325	2	0.417	8	0.314	6
Rpl13a	4.121	5	0.285	1	0.448	10	0.327	7
Eef2	6.840	8	0.740	8	0.249	5	0.344	8
Ywhaz	8.143	9	0.872	10	0.312	6	0.371	9
Tubb4b	9.322	10	0.858	9	0.430	9	0.403	10
Actb	11.000	11	1.084	11	0.609	11	0.448	11

Table S5: Stability values and rankings for the 2-hit LPS + MV study

	combined ranking		BestKeeper		NormFinder		geNorm	
gene	geometric mean of ranking value	rank	stability value	rank	stability value	rank	M value	rank
Actb	2.759	2	0.604	3	0.618	7	0.196	1
B2m	3.634	4	0.618	4	0.558	6	0.303	2
Rpl13a	3.634	4	0.549	2	0.669	8	0.339	3
Rps29	2.520	1	0.542	1	0.510	4	0.374	4
Gapdh	2.924	3	0.758	5	0.233	1	0.462	5
Eef2	4.160	6	0.848	6	0.339	2	0.511	6
Hprt	6.257	8	0.895	7	0.515	5	0.561	7
Ywhaz	5.769	7	1.052	8	0.463	3	0.603	8
Tubb4b	9.000	9	1.379	9	0.827	9	0.705	9
Tbp	10.000	10	1.502	10	0.855	10	0.782	10
Sdha	11.000	11	1.766	11	1.287	11	0.892	11

Table S6: Stability values and rankings for the 2-hit Acid + MV study

	combined ranking		BestKeeper		NormFinder		geNorm	
gene	geometric mean of ranking value	rank	stability value	rank2	stability value3	rank4	M value	rank5
Rpl13a	2.154	1	0.220	1	0.211	10	0.134	1
Rps29	3.302	3	0.241	2	0.201	9	0.136	2
Hprt	2.466	2	0.277	5	0.073	1	0.149	3
Tbp	3.302	4	0.269	3	0.118	3	0.160	4
B2m	3.915	5	0.298	6	0.093	2	0.174	5
Eef2	6.649	8	0.337	7	0.168	7	0.182	6
Sdha	8.243	10	0.406	10	0.169	8	0.193	7
Tubb4b	6.604	7	0.378	9	0.142	4	0.198	8
Gapdh	5.646	6	0.274	4	0.150	5	0.205	9
Ywhaz	7.830	9	0.360	8	0.165	6	0.213	10
Actb	11.000	11	0.460	11	0.321	11	0.237	11

Figures

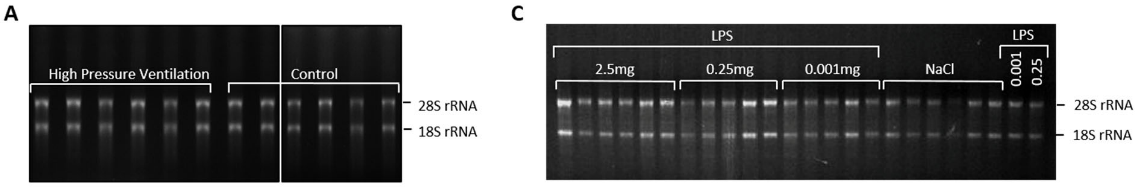

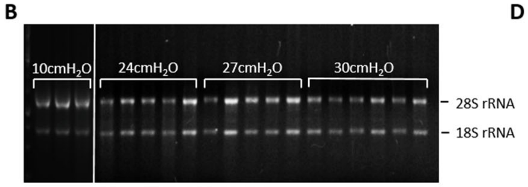

D

	NaCl	pH=2.0	pH=1.8	pH=1.5
RIN	8.3	8.7	8.4	8.2
	7.0	6.6	6.8	6.9
	7.2	7.0	6.7	7.2
	6.7	6.8	6.5	6.3
	7.1	7.2	7.3	6.9
	6.7	8.4	6.2	7.4

Figure S1: RNA integrity testing. (A) IPL study. Denaturing agarose gel electrophoresis. RNA samples were prepared with 2x RNA loading dye (Thermo Fisher Scientific; Waltham, Massachusetts, USA) and denatured by heating for 10 min at 70°C. Electrophoresis was performed in 1x MOPS buffer (0.02 morpholinopropanesulphonic acid, 5 mM sodium acetate, 0.5 mM EDTA, pH 7.0) at a voltage of 6V/cm2 for 40 min. (B) VILI and (C) LPS + MV studies: Non-denaturing agarose gel electrophoresis. RNA samples were mixed with 6x Orange DNA loading dye (Thermo Fisher Scientific; Waltham, Massachusetts, USA) and applied to a 1.5 % agarose gel containing 1x TAE buffer and GelRed (BioTrend, Cologne, Germany). Samples were separated at 150V for 90 min. Signals were visualized by the Gel DocTM XR+ Gel Documentation System (Bio-Rad Laboratories GmbH, Munich, Germany). (D) For the Acid + MV study, Bioanalyzer-assisted measurement of RNA integrity depicted as RIN values of the samples used.

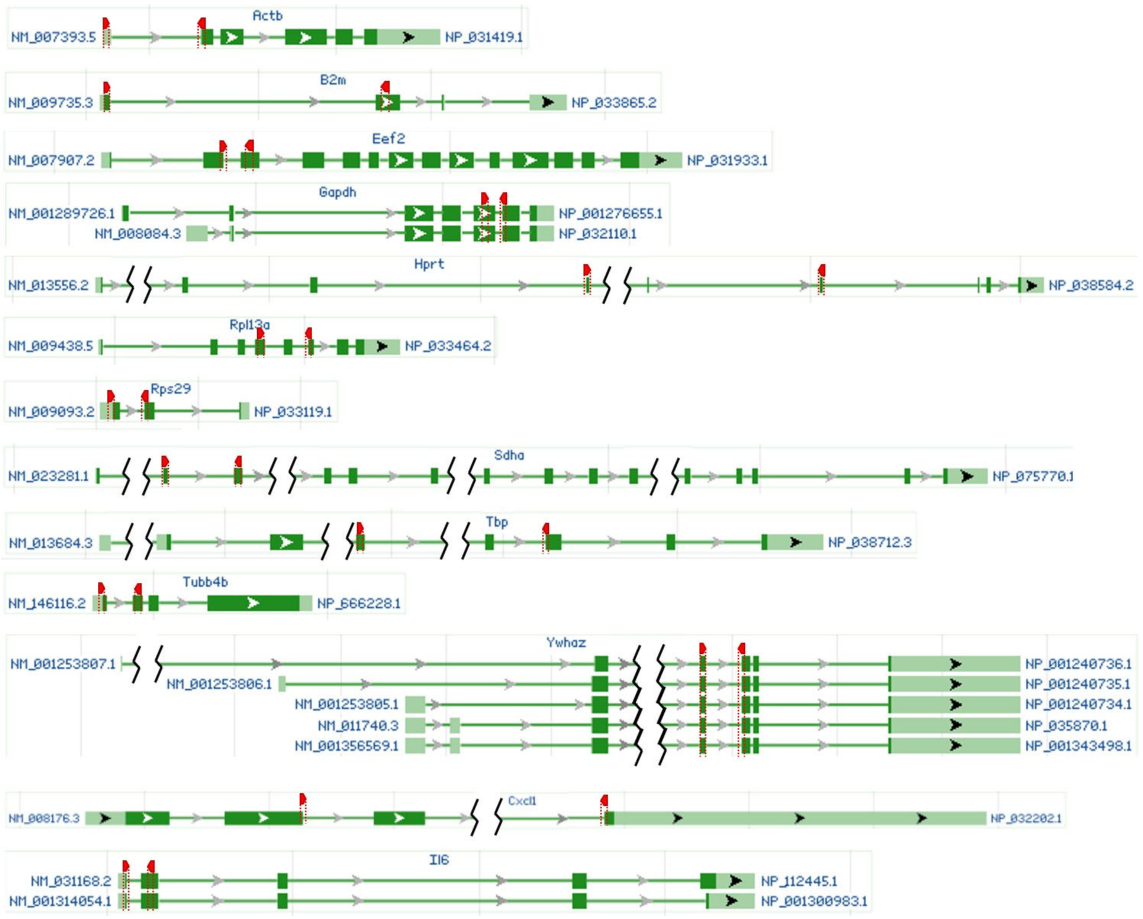

Figure S2: Primer design scheme indicating primer binding sites of the included transcripts. Exons are shown as green boxes, while UTR regions are light green instead of dark green. Introns are presented as lines with arrowheads (longer introns are interrupted to improve clarity of the depiction). Primer pairs are shown as red arrowheads with dotted lines indicating binding on the transcript as well as splice variants.

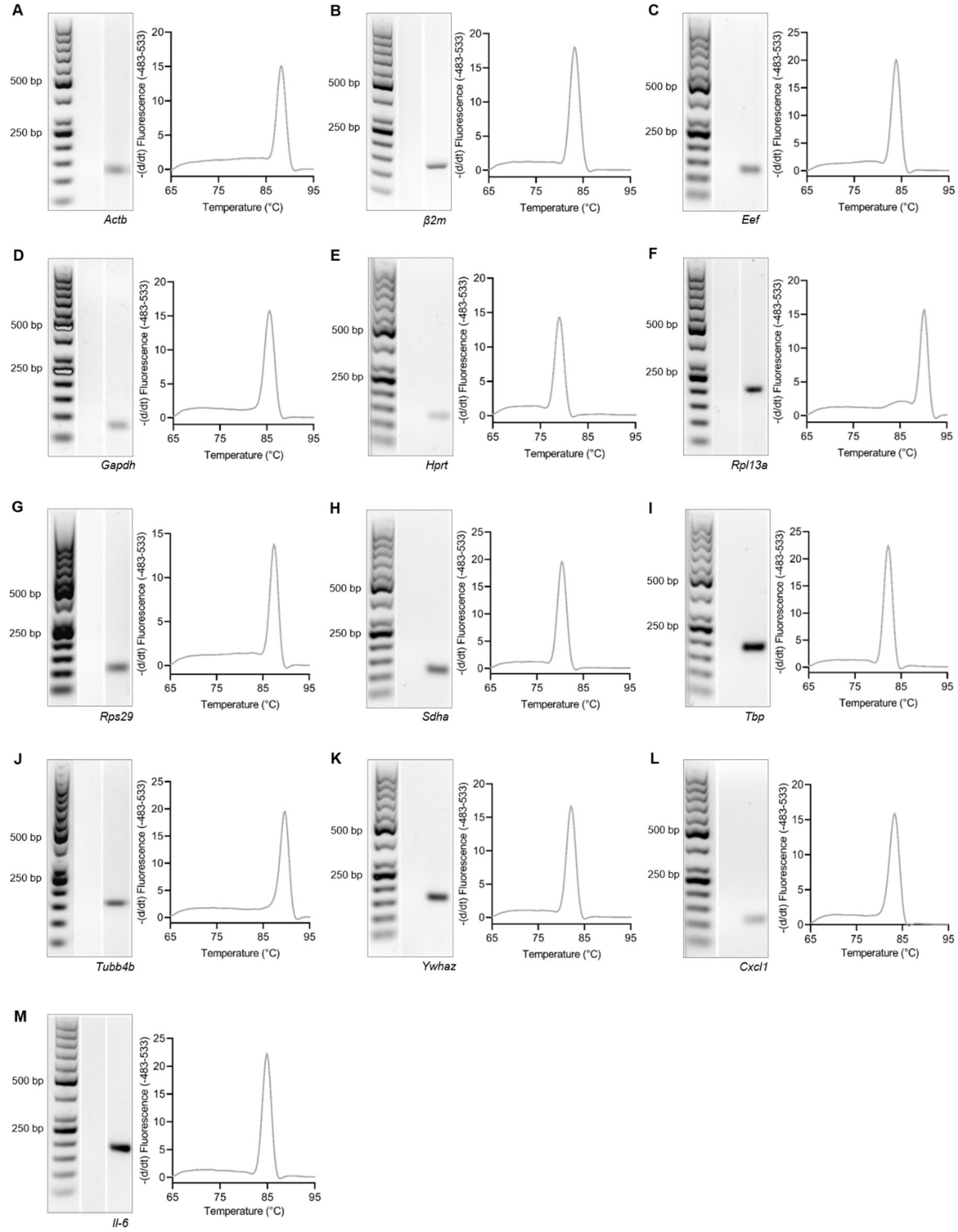

Figure S3: Melt curve analyses and gel electrophoretic separation of qPCR amplicons. Primer specificity was validated by agarose gel electrophoresis and melting curve analysis. Results are shown in alphabetical order, A-K, reference genes, L and M target genes. Left lane: DNA ladder, middle lane: nuclease-free water as negative control, right line: exemplary qPCR product with respective gene name indicated below. On the right a melting curve of one exemplary amplicon is shown. Amplicon length and melting temperature for each primer set are summarized in Table 1.
